# Oseltamivir Resistance in Adult Oncology and Hematology Patients Infected with Pandemic (H1N1) 2009 Virus, Australia

**DOI:** 10.3201/eid1607.091691

**Published:** 2010-07

**Authors:** Adrian R. Tramontana, Biju George, Aeron C. Hurt, Joseph S. Doyle, Katherine Langan, Alistair B. Reid, Janet M. Harper, Karin Thursky, Leon J. Worth, Dominic E Dwyer, C. Orla Morrissey, Paul D.R. Johnson, Kirsty L. Buising, Simon James Harrison, John F. Seymour, Patricia E. Ferguson, Bin Wang, Justin T. Denholm, Allen C. Cheng, Monica Slavin

**Affiliations:** Author affiliations: Peter MacCallum Cancer Centre, East Melbourne, Victoria, Australia (A.R. Tramontana, K. Thursky, L.J. Worth, S.J. Harrison, J.F. Seymour, M. Slavin);; Western Hospital, Footscray, Victoria, Australia (A.R. Tramontana);; Westmead Hospital, Sydney, New South Wales, Australia (B. George, D.E. Dwyer, B. Wang);; World Health Organization Collaborating Centre for Reference and Research on Influenza, North Melbourne, Victoria, Australia (A.C. Hurt);; The Alfred Hospital, Prahran, Victoria, Australia (J.S. Doyle, C.O. Morrissey);; Austin Health, Heidelberg, Victoria, Australia (K. Langan, P.D.R. Johnson);; St. Vincent’s Hospital, Melbourne, Victoria, Australia (A.B. Reid, K.L. Buising);; Royal Melbourne Hospital, Melbourne (J.M. Harper, J.T. Denholm, M. Slavin);; Centre for Infectious Diseases and Microbiology, Sydney (P.E. Ferguson);; Monash University, Prahran (A.C. Cheng)

**Keywords:** Influenza A virus, H1N1 subtype, oseltamivir, drug resistance, viruses, hematologic malignancies, hematopoietic stem cell transplantation, immunocompromised host, influenza, synopsis

## Abstract

We describe laboratory-confirmed influenza A pandemic (H1N1) 2009 in 17 hospitalized recipients of a hematopoietic stem cell transplant (HSCT) (8 allogeneic) and in 15 patients with malignancy treated at 6 Australian tertiary centers during winter 2009. Ten (31.3%) patients were admitted to intensive care, and 9 of them were HSCT recipients. All recipients of allogeneic HSCT with infection <100 days posttransplantation or severe graft-versus-host disease were admitted to an intensive care unit. In-hospital mortality rate was 21.9% (7/32). The H275Y neuraminidase mutation, which confers oseltamivir resistance developed in 4 of 7 patients with PCR positive for influenza after >4 days of oseltamivir therapy. Three of these 4 patients were critically ill. Oseltamivir resistance in 4 (13.3%) of 30 patients who were administered oseltamivir highlights the need for ongoing surveillance of such resistance and further research on optimal antiviral therapy in the immunocompromised.

## Abstract 2

Resistance in virus-infected stem cell transplant recipients illustrates the need for surveillance.

Immunocompromised patients are at risk for serious complications from seasonal influenza (*1*). This group of patients has also been disproportionately represented among those with severe infections from influenza A pandemic (H1N1) 2009, comprising 3.4%–19.6% of patients admitted to intensive care units in case series from North America (*2**–**4*).

The protective effect of seasonal influenza vaccination is reduced in patients with hematologic malignancy and recipients of an allogeneic hematopoietic stem cell transplant (HSCT) (*5**,**6*). Therefore, these patients are likely to remain at increased risk for complications from the pandemic (H1N1) 2009 virus, despite the availability of an effective vaccine. Furthermore, the emergence of resistance to neuraminidase inhibitors may limit the utility of prophylaxis in this population (*7**–**12*).

During 2009, the predominant strain of influenza in Australia was influenza A pandemic (H1N1) 2009 virus (*13**,**14*). In the state of Victoria, after the initial 3 months of community transmission of pandemic (H1N1) 2009 virus, 50% of patients who died had an underlying hematologic malignancy (*15*). We describe in detail the clinical features, treatment, and outcomes of immunocompromised patients hospitalized in 6 tertiary centers in Australia during winter 2009.

## Study Design and Population

Six Australian tertiary centers, 5 in Melbourne, Victoria, and 1 in Sydney, New South Wales (NSW), participated in the study. The 5 centers in Melbourne provide most specialist adult hematology services to the state of Victoria (population 5.4 million), and 2 of these centers perform all allogeneic HSCTs for the state. The participating Sydney center is the largest of 2 centers that perform adult allogeneic HSCT for the state of NSW (population 7 million). Approval for this study was obtained from human research ethics committees of each center.

Patients were included in the study if they had the following characteristics: 1) >18 years of age hospitalized at 1 of the 5 Melbourne study centers during May 1–August 30, 2009, or hospitalized at the Sydney center during May 1–September 15, 2009; 2) recipient of an HSCT or had an underlying malignancy (hematologic or solid tumor); and 3) had laboratory-confirmed pandemic (H1N1) 2009 virus infection identified by nucleic acid testing (NAT) during their hospital stay. At each center, investigators were directly involved in active surveillance and management of patients with pandemic (H1N1) 2009, allowing cases to be identified. Data were retrospectively abstracted onto standardized case record forms.

## Influenza Virus Diagnostics

All cases were confirmed in laboratories whose performance is accredited by the National Association of Testing Authorities in Australia. For the Victorian cases, all but 1 case-patient had laboratory confirmation of pandemic (H1N1) 2009 at the Victorian Infectious Diseases Reference Laboratory (VIDRL) with pandemic (H1N1) 2009–specific NAT by using reverse transcriptase–PCR (RT-PCR) as described (*16*). NAT for the other case-patient from Victoria was performed by the laboratory at the hospital where the patient was treated. The NSW cases were all confirmed at the state’s reference laboratory with RT-PCR assays as previously described (*17*). Patient specimens had repeat NAT at the discretion of the treating clinicians. Virus isolates or clinical samples from patients whose NAT results were positive after 4 days of oseltamivir therapy were analyzed to determine the presence of the H275Y neuraminidase (NA) mutation (N1 numbering) by using either a pyrosequencing assay (Biotage AB, Uppsala, Sweden) according to the manufacturer’s instructions, or rolling circle amplification (*18*). The H275Y mutation confers high-level oseltamivir resistance and has been detected in all of the oseltamivir-resistant pandemic (H1N1) 2009 viruses reported to date, as well as in local circulating seasonal influenza A (H1N1) strains (*8**,**12*). Samples also underwent NAT for other respiratory viruses by using a multiplex PCR.

## Definitions and Statistics

Data were stored in Microsoft Access 2003 (Microsoft, Redmond, WA, USA), and descriptive statistics were summarized with proportional outcomes. Nosocomial acquisition was defined as the development of symptoms attributable to pandemic influenza after 48 hours in the hospital that were not present on admission. Steroid-refractory or grade III–IV graft-versus-host-disease (GVHD) was defined as severe GVHD.

Length of stay was calculated as length of hospital admission or period of confinement after onset of symptoms for those with nosocomial acquisition. Epidemic curves for the 2 states were aligned with epidemic curves of community pandemic influenza activity ascertained from surveillance data (*14**,**19*). Daily corticosteroid doses were calculated by using relative glucocorticoid potency to convert to prednisolone equivalents, and mean daily values were determined for patients who received regular intermittent dosing (*20*).

Thirty-two patients fulfilled the inclusion criteria. Patient demographics and clinical features are summarized in Table 1. Seven (21.7%) of the 32 patients died; median length of stay was 6.5 d (interquartile range 4.0–13.5 d).

**Table 1:** Demographics and clinical findings among patients who had malignancy or HSCT and influenza A pandemic (H1N1) 2009 virus, Australia*

Patient no.	Age, y/sex	Underlying malignancy	Coexisting conditions	Acquisition	Radiographic infiltrates	ICU	Death
Allogeneic stem cell transplant recipients							
1	44/F	Prolymphocytic leukemia	GVHD, renal	Community†	None	Yes	Yes
2	53/F	Non-Hodgkin lymphoma	GVHD‡	Nosocomial	Multifocal	Yes	Yes
3	33/M	Hodgkin lymphoma	None	Community	None	No	No
4	57/F	CML	GVHD§	Community	Unifocal	No	No
5	61/M	Myelodysplastic syndrome	None	Nosocomial	Multifocal	Yes	Yes
6	56/M	Myelofibrosis	GVHD‡	Community	Multifocal	Yes	Yes
7	61/M	AML	GVHD,§ cardiac	Community	None	No	No
8	63/F	AML	None	Nosocomial	Unifocal	Yes	No
Autologous stem cell transplant recipients							
9	70/M	Multiple myeloma	Pulmonary	Community†	Unifocal	Yes	No
10	50/F	Multiple myeloma	None	Community	Multifocal	Yes	Yes
11	72/M	Multiple myeloma	Type 2 diabetes	Community	None	No	No
12	42/F	Multiple myeloma	Type 2 diabetes	Community	Multifocal	Yes	No
13	57/M	Multiple myeloma	None	Community†	Multifocal	No	No
14	30/F	Hodgkins lymphoma	None	Community	Multifocal	No	No
15	52/F	Non-Hodgkins lymphoma	None	Community	None	No	No
16	68/M	Multiple myeloma	Renal	Community	Multifocal	Yes	Yes
Patients with no prior stem cell transplant							
17	24/F	Hodgkins lymphoma	None	Community¶	None	No	No
18	72/M	AML	Type 2 diabetes	Community	Multifocal	No	No
19	63/F	Multiple myeloma	None	Community	Multifocal	No	No
20	80/F	Aplastic anemia	Cardiac	Community	None	No	No
21	70/F	Hodgkins lymphoma	None	Community	None	No	No
22	61/F	CLL	None	Community†	Multifocal	No	No
23	63/F	Non-Hodgkin lymphoma	Pulmonary	Community†	None	No	No
24	68/M	CLL	Pulmonary	Community	None	Yes	Yes
25	76/M	CLL	Pulmonary	Community	Multifocal	No	No
26	59/F	AML	None	Community	None	No	No
27	47/F	CLL	None	Community	No imaging	No	No
28	57/M	Multiple myeloma	None	Community	Unifocal	No	No
29	56/M	Hodgkin lymphoma	Type 2 diabetes	Community	Multifocal	No	No
30	26/M	ALL	None	Nosocomial	No imaging	No	No
31	55/F	Cervical cancer	None	Community	None	No	No
32	50/F	Breast cancer	None	Community	Multifocal	No	No

*HSCT, hematopoietic stem cell transplant; ICU, intensive care unit; GVHD, graft-versus-host disease; CML, chronic myeloid leukemia; AML, acute myeloid leukemia; CLL, chronic lymphocytic leukemia; ALL, acute lymphocytic leukemia. †Household contacts with influenza-like illness identified. ‡Severe GVHD. §Chronic GVHD. ¶Patient 17 had onset of influenza-like symptoms on returning to Victoria from interstate travel.

## Patient Demographics and Baseline Features

Eight patients were recipients of an allogeneic HSCT (Table 1). Six patients had a human leukocyte antigen (HLA) matched related, 1 (patient 1) an HLA mismatched related, and 1 (patient 7) an HLA matched unrelated allogeneic HSCT. Patient 6 was the recipient of 2 allogeneic HSCTs. Three patients (5, 6, and 8) were diagnosed with pandemic (H1N1) 2009 within 100 days of the allogeneic HSCT. Six were receiving calcineurin inhibitors. Patient 3 was the only allogeneic HSCT recipient that had documented recurrence of underlying disease posttransplant.

At the time they sought treatment, all 8 recipients of autologous HSCT were >2 years posttransplant. These patients had their malignancy diagnosed between 5 and 8 years before pandemic (H1N1) 2009, except patient 11, who had multiple myeloma that had been diagnosed 3 years previously. All autologous HSCT recipients had a relapse of malignancy after transplantation, and 7 of these patients were continuing to receive active treatment for malignancy.

Two patients (26 and 29) who had had recent a diagnosis of malignancy were in remission and continuing on the primary chemotherapy treatment plan. Seven nontransplant recipients had either not shown a response or were deemed by their treating clinicians to be refractory to their current treatments. Patient 17 had an influenza-like illness (ILI) before admission for chemotherapy and autologous HSCT (further details below). Sixteen patients (3 allogeneic HSCT recipients, 6 autologous HSCT recipients, and 10 nontransplant recipients) were receiving corticosteroids before the onset of pandemic (H1N1) 2009 infection. The mean dose in prednisolone equivalents was 40.9 mg/d (range 5.0–156 mg/d).

## Findings at Initial Visit

Clinical features at patients’ initial visit coincided with peak activity of pandemic (H1N1) 2009 in the Victorian and NSW communities (Figure; Technical Appendix). Patient 17 acquired the infection during interstate travel and sought treatment in late August, 2 days after return to Melbourne. Four patients had contact with family members with ILI, and 4 patients had nosocomial acquisition without an identifiable primary or index case (Table 1).

**Figure:**
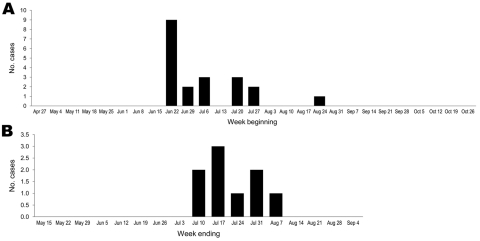
Date of admission to hospitals in Victoria (A) and New South Wales (B), Australia, for patients with underlying malignancy who were infected with pandemic (H1N1) 2009, April–October 2009. Twelve Victorian and 4 New South Wales patients were recipients of a hematopoietic stem cell transplant. Rates of laboratory detection of all influenza viruses, obtained from population-based epidemic surveillance for Victoria and New South Wales, are given in the Technical Appendix).

Most patients exhibited fever (94%) and cough (91%). Dyspnea, sore throat, and rhinorrhea were reported in 53%, 50%, and 50% of patients, respectively. Sixteen patients (50%) sought treatment within 48 h of symptom onset. Four patients visited the hospital after experiencing prolonged symptoms: for 12 d (patient 4), 14 d (patients 22 and 25), and 21 d (patient 14). Three of these patients had biphasic illnesses with initial prolonged mild upper respiratory tract illnesses and, on examination, had consolidation shown on chest radiograph. Patient 25 had 2 weeks of dyspnea associated with an exacerbation of chronic obstructive pulmonary disease. Patient 16 sought treatment for shock and respiratory failure.

Diagnosis of pandemic (H1N1) 2009 was confirmed by NAT of bronchial washings of 3 patients (2, 16, 17). One of them, patient 17, had symptoms of a viral respiratory tract infection for 20 d with negative results for pandemic (H1N1) 2009 virus by RT-PCR of nasopharyngeal swab specimens taken 2 and 16 d before bronchoscopy. All other patients had the diagnosis confirmed on NAT of nasopharyngeal swab samples. Six patients (19%) had a baseline peripheral blood lymphocyte count <200 cells/mL, and 18 (56%) patients had pneumonia shown on baseline chest radiograph.

## Antiviral Therapy

Thirty patients (94%) were administered oseltamivir therapy. Of these patients, 13 (41%) were administered therapy within 48 hours, and 16 began therapy >48 hours after symptom onset. Duration of illness before receiving oseltamivir was unknown for patient 2. Nine patients received NA inhibitors for >5 days. Three of these patients (1, 12, 18) had a positive NAT result >5 days after oseltamivir treatment began. Antimicrobial drug treatment was changed to zanamir for patients 1 and 8 after they received initial therapy with oseltamivir (see below). The longest duration of antiviral therapy was 43 d (patient 1). Patients 15 and 31 had uncomplicated infections and were not administered oseltamivir therapy.

## Co-pathogens

Four patients had notable co-pathogens identified. Patient 2 had 4 concurrent herpesviridae (cytomegalovirus, Epstein-Barr virus, herpes simplex virus 2, and human herpesvirus 6) detected by NAT in respiratory specimens and blood. Treatment for patient 2 included liposomal amphotericin B and foscarnet. Patient 7 had *Staphylococcus aureus* bacteremia from an unrelated source. Patient 17, who came to transplant with an ILI, had bronchial washings that were positive for pandemic (H1N1) 2009 by NAT, galactomannan antigen, and *Aspergillus* spp. by NAT 5 days after transplant. Pulmonary nodules consistent with invasive fungal infection were seen on a high-resolution computed tomography scan. Patient 13 was infected with respiratory syncytial virus, which was detected by multiplex NAT on a nasopharyngeal swab.

## Patients Admitted to Intensive Care Unit

Ten (31.3%) patients were admitted to intensive care (Table 1). In each case, the primary reason for admission to intensive care was respiratory failure. Seven (70%) of these patients died, 6 in intensive care and 1 (patient 5) of recurrent pneumonia after being discharged from intensive care. Preterminal events included progressive respiratory failure (n = 5) and multiorgan failure (n = 2).

All allogeneic HSCT recipients with the following features required admission to intensive care for mechanical ventilation: transplantation within 100 d, severe GVHD, and nosocomial acquisition of pandemic (H1N1) 2009. However, onset of symptoms for patient 1 was day 119 after allogeneic transplantation.

Eight of 10 patients admitted to intensive care had evidence of pneumonia on baseline chest radiograph. Patient 1 initially had normal chest radiograph results, despite the acute onset of hypoxia. Patient 24 was transferred to intensive care after 3 days in the hospital, at which point bilateral infiltrates were seen on chest radiograph, and oseltamivir therapy was begun.

## Oseltamivir Resistance

Ten patients had repeat NAT testing to determine clearance of viral shedding. Eight had >1 further positive NAT (2 on sputum, 5 on nasopharyngeal swab specimen, and 1 on bronchoalveolar lavage sample) after receiving oseltamivir. Five of these patients had a positive NAT after >5 d of oseltamivir therapy. The longest recorded duration of viral shedding during oseltamivir therapy was 28 d (patient 1).

The H275Y NA mutation, a substitution known to confer a high level of oseltamivir resistance, was detected in 4 (57%) of 7 patients who had detectable nucleic acid after >4 d of oseltamivir therapy. These 4 patients comprised 13.3% of the 30 patients who received oseltamivir. The findings for the 4 patients who were infected with oseltamivir-resistant influenza virus are summarized in Table 2. The H275Y mutation was undetectable in initial diagnostic samples from these patients. Additionally, the H275Y mutation was detected in all available samples collected from these patients after they received oseltamivir therapy. Three of the 4 patients who had oseltamivir-resistant pandemic (H1N1) 2009 virus infection were HSCT recipients who had been admitted to intensive care. Virus isolation in MDCK cells was attempted for the samples that contained the H275Y mutation but was unsuccessful after 2 passages. This precluded the use of the phenotypic NA inhibition assay to further analyze the samples.

**Table 2:** Characteristics of 4 patients infected with oseltamivir-resistant pandemic influenza A (H1N1) virus isolates, Australia*†

Characteristic	Patient no.			
	1	5	12	20
Within 100 days of HSCT‡	No	Yes	No	–
Time to development of resistance, d	22	11§	8	4
Time of last positive NAT result, d	28	16	8	4
Change to zanamivir	Yes	No	No	No
Time to zanamivir, d	36	–	–	–
Died	Yes	Yes	No	No
LOS, d	39	66	21	9

*HSCT, hematopoietic stem cell transplant; NAT, nucleic acid test; LOS, length of stay. †Oseltamivir resistance was influenza virus with H275Y mutation. ‡Time from commencement of oseltamivir. §Detected in bronchoalveolar lavage specimen with negative NAT on nasopharyngeal swab 3 d before first and 10 d after last bronchoscopy. This patient received oseltamivir for 5 d.

Patient 12, who survived despite shedding oseltamivir-resistant pandemic (H1N1) 2009 virus, had a biphasic clinical course. She initially stabilized while she was treated with oseltamivir; progressive respiratory failure then developed, which coincided with her recovery from neutropenia before later improvement. The other patient who survived despite shedding oseltamivir-resistant 2009 pandemic (H1N1) virus (patient 20) had an ILI without pneumonia. Of the 2 patients with resistant isolates who died, patient 5 first experienced the apparent resolution of pneumonia but later succumbed to recurrent pneumonia, and patient 1 had persisting pneumonitis despite 36 days of oseltamivir therapy.

Patient 24 had multiple positive NATs, none with the H275Y mutant detected. His NAT was positive 4 d after he began oseltamivir therapy, and he died 1 d after his last specimen, a nasopharyngeal swab, was collected.

## Conclusions

We report a case series of hospitalized cancer patients with influenza A pandemic (H1N1) 2009 virus infection and their outcomes. Patients with hematologic malignancies accounted for 50% of deaths of persons with pandemic influenza in Victoria during the first 3 months of the pandemic (*15*). The strongest effects of illness from pandemic influenza among hospitalized cancer patients in the present series occurred in HSCT recipients. Nine of the 10 cancer patients admitted to intensive care were HSCT recipients. Furthermore, of 7 deaths from pandemic (H1N1) 2009 in this series of hospitalized cancer patients, 6 occurred in HSCT recipients (comprising 37.5% of these patients). Our observations are similar to those seen with seasonal influenza. In a series of hematology patients with respiratory virus infection, including seasonal influenza, from 1 large cancer center, the largest number of infections and deaths occurred in recipients of allogeneic HSCTs (*21*). Our observations support the importance of existing recommendations for control of transmission of influenza infection in HSCT recipients during an influenza pandemic (*22*).

Half of the 24 cancer patients who had not received an allogeneic HSCT had underlying multiple myeloma (n = 8) and chronic lymphocytic leukemia (n = 4). There was also a paucity of patients with solid tumors (n = 2). The severity of illness from influenza in patients who have underlying multiple myeloma and chronic lymphocytic leukemia has not previously been widely appreciated, although a high rate of pneumonic illness from influenza has previously been demonstrated at 1 center (*21*). Patients with multiple myeloma have impaired cell-mediated immunity, in addition to humoral immune deficits (*6**,**23**,**24*). All 4 patients with multiple myeloma admitted to intensive care had previously received an autologous HSCT, which likely further compromised immunologic function. They also had been diagnosed with myeloma for several years.

Bacterial co-pathogens that cause pneumonia were not identified in our study cohort. However, patients received broad-spectrum antibacterial agents on admission. In contrast, a small number of investigators have identified bacterial superinfections in up to 30% of fatal cases of pandemic influenza (*3**,**25*). For patient 17 in this series, a diagnosis of pulmonary aspergillosis was made after autologous HSCT, an association previously reported with seasonal influenza in allogeneic HSCT recipients (*26*).

When there is a circulating strain of influenza not contained in the recent influenza vaccine, postexposure oseltamivir prophylaxis is an attractive strategy that can prevent the development of influenza infection (*27**–**29*). Postexposure prophylaxis of healthcare workers and family members may reduce the likelihood of exposure through prevention of influenza infection in close contacts of HSCT recipients. Our observations that would support post-exposure oseltamivir prophylaxis are 1) 4 of 5 allogeneic HSCT recipients who died or were admitted to intensive care had either nosocomial or household acquisition; 2) 5 of 11 HSCT recipients who had pneumonia before beginning oseltamivir therapy died; 3) none of the patients in this study were known to have received postexposure prophylaxis at the time of symptom onset, despite use of this strategy during the study period (*29*); and 4) 3 patients acquired the infection in the hospital from unidentified sources. This nosocomial transmission occurred despite heightened awareness during this pandemic, which demonstrates the difficulties of effective containment in the hospital setting (*28**,**29*).

The finding of oseltamivir-resistant influenza virus in 4 of 7 patients with a positive pandemic (H1N1) 2009–specific RT-PCR result after >4 d of oseltamivir therapy is a cause for concern. In Australia, oseltamivir-resistant pandemic (H1N1) 2009 has been described in another immunocompromised patient, a renal transplant recipient (*30*). No oseltamivir-resistant pandemic (H1N1) 2009 strains have been detected in nonhospitalized patients in Australia (B. Wang and A. Hurt, pers. comm.). The risk of pandemic influenza developing the H275Y mutation that confers oseltamivir resistance is considered to be higher in immunocompromised patients (*10**,**12*). Immunocompromised patients have also been overrepresented in deaths associated with oseltamivir-resistant seasonal influenza (*31**,**32*). If repeat NAT on respiratory specimens had been routinely performed for all treated patients in the present series, rates of oseltamivir resistance may have been even higher than we observed. Our findings indicate an urgent need to optimize oseltamivir dosing in immunocompromised patients. Further research into the role of combination antiviral therapy should be considered (*33**,**34*). Immunocompromised patients should have serial screening for ongoing viral shedding and oseltamivir resistance. The oseltamivir-resistant H275Y mutants remain susceptible to the alternative NA inhibitor zanamivir, and therefore the use of this drug should be considered in patients who are shedding these viruses (*10*).

Only 1 of the patients who was in intensive care and had oseltamivir-resistant pandemic (H1N1) 2009 virus survived. The effects of oseltamivir-resistant influenza virus in this patient are uncertain. The deterioration of her condition after initial stabilization may have been related to development of oseltamivir-resistance and persistent influenza viral replication similar to that seen in a recent case report (*30*). Others have shown development of resistance to oseltamivir within 3 d of therapy (*30*), and we observed resistance develop within 4 d for 1 patient. Undoubtedly, other explanations for her biphasic illness are possible. Immune recovery with resolution of neutropenia may have enabled viral clearance and recovery.

Universal chemoprophylaxis for HSCT recipients during an outbreak with an influenza strain that is not contained in the available influenza vaccine has been recommended by international guidelines supported by North American and European Blood and Marrow Transplant groups, Infectious Diseases Society of America, and the US Centers for Disease Control and Prevention (*22*). One of the study centers instituted universal chemoprophylaxis for patients admitted to receive an allogeneic HSCT after 2 cases of nosocomial-acquired infection before engraftment (P. Ferguson, pers. comm.). Although the strategy led to apparent success, with no further nosocomial infections observed, we have concern about this approach based on 1) the high rate of oseltamivir resistance (4/30 [13.3%] administered oseltamivir) observed in this study; 2) overrepresentation of postexposure prophylaxis in cases of oseltamivir-resistant pandemic (H1N1) 2009 influenza (*12*); 3) the potential that pandemic (H1N1) 2009 H275Y mutant viruses may transmit and spread throughout the community, similar to outcomes recently observed with seasonal influenza A (H1N1) viruses with the same H275Y mutation (*8**,**11**,**35*); and 4) the paucity of hospital-acquired cases seen in this series during heightened surveillance and control measures of a pandemic. A more judicious approach may be surveillance, infection control measures, and early treatment until vaccination becomes available (*12**,**22**,**28*). Additionally, education of patients and their close contacts to facilitate early treatment and avoidance of exposure is essential. When a vaccine becomes available, close contacts of those infected, their family members, and healthcare workers should be vaccinated.

One of the strengths of this study is the relatively high coverage of the major hematology centers in a single state, which reduces the effect of bias on patient ascertainment. However, patients whose treatment was managed in the community were excluded, and this information will be essential to our understanding as to which co-factors predict clinical progression and outcomes for pandemic (H1N1) 2009 infection in immunocompromised patients.

A high rate of oseltamivir resistance developed in critically ill HSCT recipients, particularly in those who continued to shed pandemic (H1N1) 2009 virus after 4 d of oseltamivir treatment. Consequently, surveillance, infection control measures, and early treatment of those at greatest risk of pandemic (H1N1) 2009 infection may prove more useful than universal chemoprophylaxis during an outbreak with an influenza strain that is not contained in the available influenza vaccine. Continued surveillance for oseltamivir-resistant influenza virus strains is needed, particularly in the immunocompromised.

## Supplementary Material

Technical Appendix Oseltamivir Resistance in Adult Oncology and Hematology Patients Infected with Pandemic (H1N1) 2009 Virus, Australia
