# Supplementary material for: Oseltamivir Resistance in Adult Oncology and Hematology Patients Infected with Pandemic (H1N1) 2009 Virus, Australia
Source: Emerg Infect Dis. 2010 Jul;16(7):1068–75. doi: 10.3201/eid1607.091691 (PMC3321901; doi:10.3201/eid1607.091691)
Supplement: Technical Appendix — Oseltamivir Resistance in Adult Oncology and Hematology Patients Infected with Pandemic (H1N1) 2009 Virus, Australia [file 09-1691-Techapp.pdf]

DOI: 10.3201/eid1607.091691

Oseltamivir Resistance in Adult Oncology and Hematology Patients Infected with Pandemic (H1N1) 2009 Virus, Australia

Technical Appendix

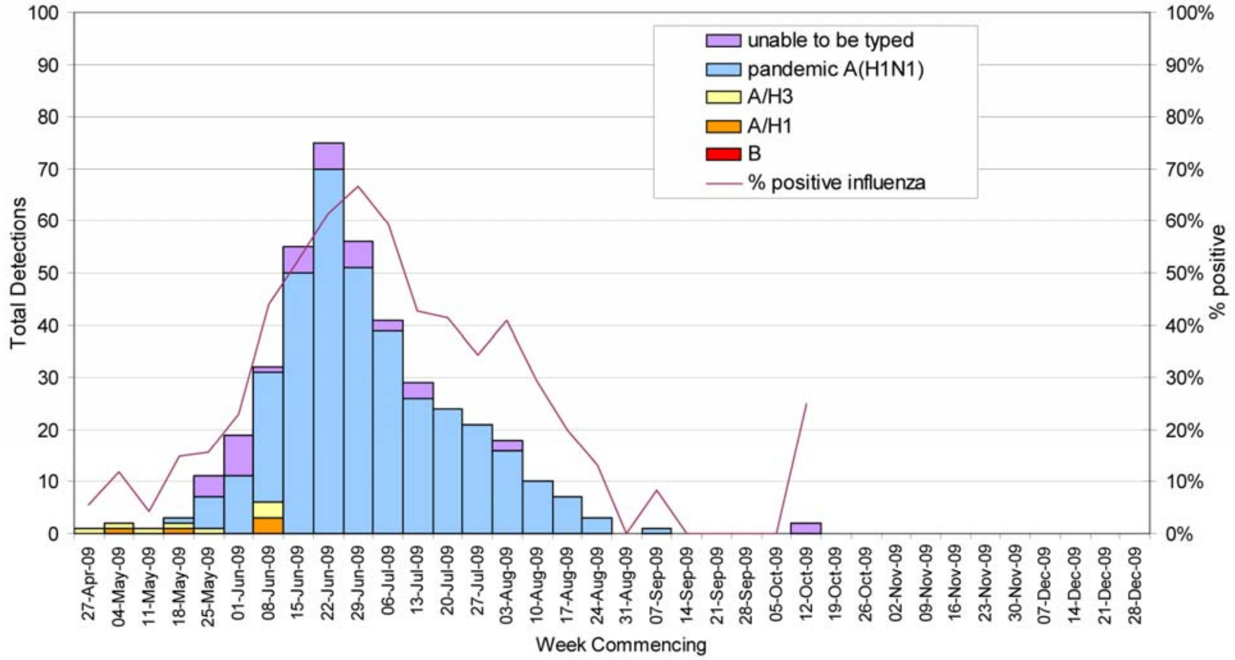

Laboratory detection of influenza viruses from general practitioner sentinel surveillance for weeks starting April 27–December 28, 2009, Victoria, Australia. Obtained with permission from www.vidrl.org.au/surveillance/flu%20reports/flurpt09.html.

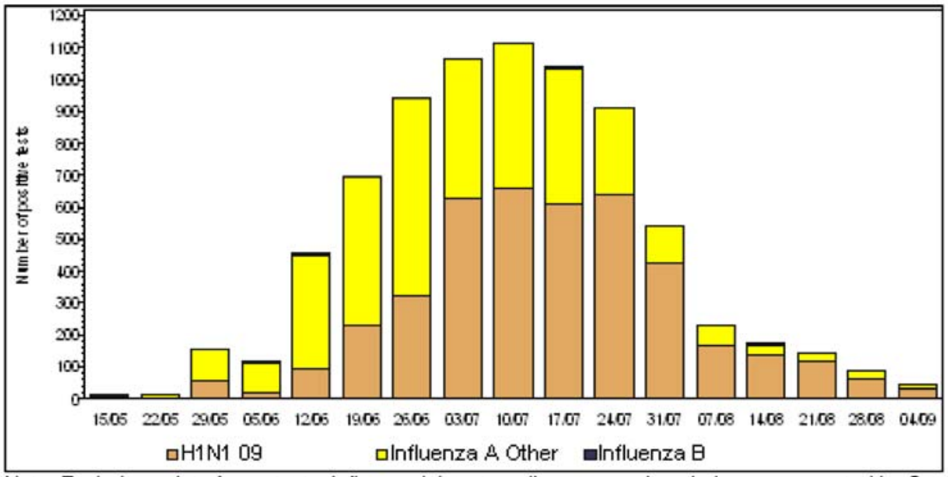

Note: Excludes point of care tests. Influenza laboratory diagnoses using virology are reported by South Eastern Area Laboratory Services (SEALS), Institute of Clinical Pathology and Medical Research (ICPMR), South West Area Pathology Services (SWAPS)- from 12/6, Pacific Laboratory Medicine Services (PaLMS)-from 19/6, Royal Prince Alfred Hospital (RPAH), Hunter Area Pathology Services (HAPS), Nepean-from 24/7 and St Vincent's Hospital (SYDPATH).

Laboratory detection of influenza viruses at selected public hospital laboratories for weeks ending May 15–September 4, 2009, New South Wales, Australia. Obtained with permission from New South Wales Health's Population Health division.
